# An enzyme complex increases in vitro dry matter digestibility of corn and wheat in pigs

**DOI:** 10.1186/s40064-016-2194-5

**Published:** 2016-05-11

**Authors:** Kyu Ree Park, Chan Sol Park, Beob Gyun Kim

**Affiliations:** Department of Animal Science and Technology, Konkuk University, Seoul, 05029 Republic of Korea

**Keywords:** Corn fractions, Exogenous enzymes, Feed ingredients, In vitro dry matter digestibility, Pigs

## Abstract

Two experiments were conducted to determine the effects of enzyme complex on in vitro dry matter (DM) digestibility for feed ingredients. The objective of experiment 1 was to screen feed ingredients that can be effective substrates for an enzyme complex, mainly consisted of β-pentosanase, β-glucanase and α-amylase, using in vitro digestibility methods. In experiment 1, the test ingredients were three grain sources (barley, corn and wheat) and six protein supplements (canola meal, copra expellers, cottonseed meal, distillers dried grains with solubles, palm kernel expellers and soybean meal). In vitro ileal and total tract digestibility (IVID and IVTTD, respectively) of DM for test ingredients were determined. In vitro digestibility methods consisted of two- or three-step procedure simulating in vivo digestion in the pig gastrointestinal tracts with or without enzyme complex. As the enzyme complex added, the IVID of DM for corn and wheat increased (*p* < 0.05) by 5.0 and 2.6 percentage unit, respectively. The IVTTD of DM for corn increased (*p* < 0.05) by 3.1 percentage unit with enzyme complex addition. As the effect of enzyme complex was the greatest in corn digestibility, corn grains were selected to determine the in vitro digestibility of the fractions (starch, germ, hull and gluten) that maximally respond to the enzyme complex in experiment 2. The IVID of DM for corn starch, germ and hull increased (*p* < 0.05) by 16.0, 2.8 and 1.2 percentage unit, respectively. The IVTTD of DM for corn starch and hull also increased (*p* < 0.05) by 8.6 and 0.9 percentage unit, respectively, with enzyme complex addition. In conclusion, the enzyme complex increases in vitro DM digestibility of corn and wheat, and the digestibility increments of corn are mainly attributed to the increased digestibility of corn starch.

## Background

Many feed ingredients for swine diets have a considerable amount of non-starch polysaccharide (NSP), which acts as an anti-nutritional factor (Bedford and Schulze 1998; Masey O’Neill et al. 2014). Inclusion of exogenous NSP-degrading enzyme in diet may increase the energy values by increasing digestibility of nutrients. Bergazyme-P^®^ is an enzyme complex that is consisted of β-pentosanase, β-glucanase, α-amylase, protease, glucanase and galactomannanase. The one of the main enzymes in this product is β-pentosanase and one of main substrates of this enzyme, arabinoxylan, is most common NSP in cereal grains (Masey O’Neill et al. 2014). Supplementation of the enzyme complex, Bergazyme-P^®^, at 0.025 and 0.050 % has been reported to improve growth performance and amino acid digestibility in broilers (Abudabos 2010). However, information on the effects of this enzyme complex to be tested in this study on pigs is very limited.

To effectively use an exogenous enzyme in swine diets, feed ingredients which greatly respond to the enzyme should be selected. In vitro digestibility methods that simulate the gastrointestinal tract of pigs have been used for the prediction of in vivo digestibility values (Boisen and Fernández 1995, 1997; Regmi et al. 2009; Park et al. 2012). Before using exogenous enzyme in swine diet, the in vitro digestibility methods can be used to determine the efficacy of exogenous enzyme (Kong et al. 2015).

Therefore, the objectives of this study were to screen the feed ingredients for the enzyme complex using in vitro digestibility methods and to determine the effects of the enzyme complex on the in vitro ileal and total tract digestibility (IVID and IVTTD, respectively) of dry matter (DM) for the fractions of an ingredient that maximally respond to the enzyme complex.

## Methods

### Enzyme complex and sample preparation

The Bergazyme-P^®^ contained mainly β-pentosanase, β-glucanase, α-amylase and protease at 6000 EPU/g, 32,000, 17,600 and 142 EU/g, respectively. The enzyme product sample was provided by Feed Best Inc. (Seoul, Republic of Korea). The test ingredients were finely grounded to pass a 1-mm screen (Cyclotech 1093; Foss Tecator AB, Höganäs, Sweden), and the ingredients were divided into two groups. Each group of samples was prepared to contain 1 % of wheat bran or 1 % of enzyme complex, respectively.

### Experiment 1: Screening of feed ingredient

Feed ingredients used for screening study were three whole grain sources (barley, corn and wheat) and six protein supplements (canola meal, copra expellers, cottonseed meal, distillers dried grains with solubles, palm kernel expellers and soybean meal). Both IVID and IVTTD of DM for the prepared samples were determined.

### Experiment 2: Effects of enzyme complex on the fractions of corn grains

The experiment 2 was conducted based on the results derived from the experiment 1. Divided fractions of corn grains (starch, germ, hull and gluten) were tested. Sample preparation and experimental procedure were identical to the experiment 1.

### In vitro ileal digestibility method

The IVID method included two-step enzymatic degradations that simulated the digestion in the stomach and small intestine based on the procedures described by Boisen and Fernández (1995).

In the first step, 1 g of sample was placed in a 100 mL conical flask, and 25 mL of phosphate buffer solution (0.1 M, pH 6.0) and 10 mL of 0.2 M HCl were added in the test flasks. Then the pH was adjusted to 2.0 using 1 M HCl or NaOH solution, and 1 mL of freshly prepared pepsin solution (10 mg/mL; ≥ 250 U/mg solid, P7000, Pepsin from porcine gastric mucosa, Sigma-Aldrich, St. Louis, MO, USA) was added. To prevent bacterial fermentation, 0.5 mL of chloramphenicol (C0378, Chloramphenicol, Sigma-Aldrich, St. Louis, MO, USA) solution (5 g/L ethanol) was added. The test flasks were closed with a silicon stopper and incubated in a shaking incubator at 39 °C for 6 h.

After the incubation, the second step of procedure was conducted. Firstly, 10 mL of phosphate buffer solution (0.2 M, pH 6.8) and 5 mL of 0.6 M NaOH solution were added in the test flasks. Then pH was adjusted to 6.8 using 1 M HCl or NaOH solution, and 1 mL of freshly prepared pancreatin solution (50 mg/mL; 4 × USP, P1750, Pancreatin from porcine pancreas, Sigma-Aldrich, St. Louis, MO, USA) was added. Then the test flasks were incubated in a shaking incubator at 39 °C for 18 h.

After the incubation, the test flasks were added 5 mL of 20 % sulfosalicylic acid solution and left at room temperature for 30 min to precipitate indigested protein. After 30 min of precipitation, undigested samples were filtered through pre-dried and weighed glass filter crucibles (Filter Crucibles CFE Por. 2, Robu, Hattert, Germany) containing 400 mg of Celite as filter aid using the Fibertec System (Fibertec System 1021 Cold Extractor, Tecator, Hӧganӓs, Sweden). The test flasks were rinsed twice by 1 % sulfosalicylic acid solution. Undigested samples in glass filter crucible were rinsed twice with 10 mL of 95 % ethanol and 10 mL of 99.5 % acetone. Glass filter crucibles with undigested samples were dried at 130 °C for 6 h. After 1 h cooling in a desiccator, glass filter crucibles were weighed.

### In vitro total tract digestibility method

The IVTTD method consisted of three-step enzymatic degradations that simulated the digestion in the stomach, small intestine and large intestine based on the procedures described by Boisen and Fernández (1997).

The first and second steps were similar to the procedures of IVID except the weight of sample, concentration of pepsin and pancreatin solutions and incubation time. For IVTTD, 0.5 g of sample was used, and the concentrations of pepsin and pancreatic solutions were increased to 25 and 100 mg/mL, respectively, while the incubation times were reduced to 2 and 4 h, respectively.

In the third step, 10 mL of 0.2 M EDTA solution was added in the test flasks. The pH was adjusted to 4.8 using 30 % of acetic acid or 1 M NaOH solution. As a substitution of microbial enzyme for simulating hind-gut microbial fermentation, 0.5 mL of Viscozyme (V2010, Viscozyme^®^ L, Sigma-Aldrich, St. Louis, MO, USA) was added. Then the test flasks were incubated in a shaking incubator for 18 h at 39 °C.

After the incubation, undigested samples were filtered in pre-dried and weighed glass filter crucibles containing 500 mg of Celite as filter aid using the Fibertec System (Fibertec System 1021 Cold Extractor, Tecator, Hӧganӓs, Sweden). The test flasks were rinsed twice by distilled water. Undigested samples in glass filter crucibles were rinsed twice with 10 mL of 95 % ethanol and 99.5 % acetone. Then, glass filter crucibles with undigested samples were dried at 130 °C for 6 h. After 1 h cooling in a desiccator, glass filter crucibles were weighed.

### Calculations and statistical analyses

The IVID and IVTTD of DM (%) were calculated with following equation:

$${\text{IVID or IVTTD of DM }} = \, \left[ {\left( {{\text{DM}}_{\text{TI}} - {\text{ DM}}_{\text{RS}} } \right)/{\text{DM}}_{\text{TI}} } \right] \, \times \, 100,$$

where DM_TI_ (g) is the weight of DM in the test ingredient and DM_RS_ (g) is weight of DM in the undigested residue collected from IVID or IVTTD procedures.

Data were analyzed by GLM procedure of SAS (SAS Inst. Inc., Cary, NC, USA). The model included the enzyme complex addition as the independent variable. Least squares means for IVID and IVTTD of DM for each ingredient with or without the enzyme complex addition were calculated. The experimental unit was a test flask and significance of treatment effects were declared at *p* < 0.05.

## Results and discussion

There are many studies that assessed the effects of various exogenous enzyme products for swine diets, but the results of these experiments were different due to the differences between studies such as the enzymes, response criteria and ingredients compositions (Kwon et al. 2015; Jones et al. 2015; Mok et al. 2015). These in vivo experiments require many expenses such as labor and money. To save the expenses, in vitro digestibility technique has been used to predict in vivo digestibility of energy and nutrients for pigs (Boisen and Fernández 1995, 1997; Noblet and Jaguelin-Peyraud 2007; Regmi et al. 2009; Park et al. 2012).

In experiment 1, the IVID of DM for corn and wheat increased (*p* = 0.029 and *p* = 0.003, respectively; Table 1) with the enzyme complex addition compared with the control. The IVTTD of DM for corn also increased (*p* = 0.002; Table 2) with enzyme complex addition. However, the IVID and IVTTD of DM for other test ingredients were not affected by enzyme complex addition. The values of in vitro DM digestibility of ingredients in other studies were fairly close to the ones obtained in the current study (Boisen and Fernández 1995, 1997; Regmi et al. 2009; Kong et al. 2015). Based on the results of experiment 1, corn was selected as an ingredient for further study, because the changes as percentage unit of the in vitro DM digestibility for corn were the largest, although the IVID of DM for wheat significantly increased with enzyme complex addition. But the NSP contents in corn are generally less than other ingredients used in current experiment (NRC 2012; Masey O’Neill et al. 2014). To identify the fraction of corn grain that maximally responded to the enzyme complex, experiment 2 was conducted with fractions of corn grains.

**Table 1:** In vitro ileal digestibility of dry matter for feed ingredients with or without the enzyme complex, experiment 1

Item	Digestibility, %		SEM	*p* value
	Control	Enzyme complex addition^a^		
Barley	75.6	77.6	0.91	0.168
Corn	75.5	80.5	1.35	0.029
Wheat	83.8	86.4	0.47	0.003
Canola meal	64.5	63.1	0.87	0.893
Copra expellers	48.5	45.9	1.88	0.351
Cotton seed meal	56.6	55.5	0.74	0.285
Distillers dried grains with solubles	62.8	61.7	0.78	0.372
Palm kernel expellers	30.7	31.6	0.39	0.162
Soybean meal	74.6	74.2	0.34	0.424

Each least squares mean represents 6 observations except corn in control and cotton seed meal with an enzyme addition (5 observations)

*SEM* standard error of the means

^a^The enzyme complex was added at 1.0 %. The enzyme complex consisted of β-pentosanase, β-glucanase, α-amylase and protease (6000 EPU/g, 32,000, 17,600 and 142 EU/g, respectively)

**Table 2:** In vitro total tract digestibility of dry matter for feed ingredients with or without the enzyme complex, experiment 1

Item	Digestibility, %		SEM	*p* value
	Control	Enzyme complex addition^a^		
Barley	85.9	86.4	0.43	0.515
Corn	88.9	92.0	0.48	0.002
Wheat	90.1	91.1	0.50	0.175
Canola expellers	78.2	77.8	0.16	0.123
Copra meal	65.7	66.5	0.55	0.334
Cotton seed meal	64.6	65.0	0.72	0.670
Distillers dried grains with solubles	75.1	73.4	0.71	0.145
Palm kernel expellers	47.3	47.0	0.68	0.775
Soybean meal	94.1	93.9	0.21	0.367

Each mean represents 6 observations except soybean meal in control and soybean meal and DDGS with an enzyme addition (5 observations)

*SEM* standard error of the means

^a^The enzyme complex was added at 1.0 %. The enzyme complex consisted of β-pentosanase, β-glucanase, α-amylase and protease (6000 EPU/g, 32,000, 17,600 and 142 EU/g, respectively)

In experiment 2, The IVID of DM for corn starch, germ and hull increased (*p* < 0.001, *p* = 0.001 and *p* = 0.015, respectively; Table 3) with enzyme complex addition. The IVTTD of DM for corn starch and hull increased (*p* = 0.011 and *p* = 0.016, respectively; Table 4) with enzyme complex addition, but corn germ and gluten were not affected by enzyme complex addition. Corn generally consisted of 64.6 % starch, 14.8 % germ, 7.7 % hulls and 5.6 % gluten (Corn Refiners Association 2006; Zilic et al. 2011). In experiment 1, most ingredients whose in vitro digestibility increased with enzyme complex have high starch contents. In addition, digestibility of corn starch had the largest value of changes as percentage unit in experiment 2. Perhaps the significant difference in high-starch test ingredients was associated with insufficient amylase concentration from pancreatin solution to digest all of starch contents in high starch test ingredients. However, the experiment employing corn fractions may not necessarily provide full picture of enzyme actions on an ingredient as the potential interactive actions between the nutrient matrix and the enzyme complex are not fully tested (Jha et al. 2015).

**Table 3:** In vitro ileal digestibility of dry matter for fractions of corn grains with or without the enzyme complex, experiment 2

Item	Digestibility, %		SEM	*p* value
	Control	Enzyme complex addition^a^		
Starch	66.5	82.6	1.3	<0.001
Germ	58.8	61.6	0.4	0.001
Hull	55.3	56.5	0.3	0.015
Gluten	64.2	59.9	1.7	0.105

Each mean represents 6 observations

*SEM* standard error of the means

^a^The enzyme complex was added at 1.0 %. The enzyme complex consisted of β-pentosanase, β-glucanase, α-amylase and protease (6000 EPU/g, 32,000, 17,600 and 142 EU/g, respectively)

**Table 4:** In vitro total tract digestibility of dry matter for fractions of corn grains with or without the enzyme complex, experiment 2

Item	Digestibility, %		SEM	*p* value
	Control	Enzyme complex addition^a^		
Starch	87.9	96.4	1.9	0.011
Germ	64.0	65.1	0.9	0.407
Hull	59.2	60.1	0.2	0.016
Gluten	64.4	64.7	1.0	0.862

Each mean represents 6 observations except gluten with an enzyme addition (5 observations)

*SEM* standard error of the means

^a^The enzyme complex was added at 1.0 %. The enzyme complex consisted of β-pentosanase, β-glucanase, α-amylase and protease (6000 EPU/g, 32,000, 17,600 and 142 EU/g, respectively)

In conclusion, the enzyme complex increases in vitro DM digestibility for corn and wheat, and the digestibility increments of corn are mainly attributed to the increased digestibility of corn starch.
